# Effect of pesticides on microbial communities in container aquatic habitats

**DOI:** 10.1038/srep44565

**Published:** 2017-03-16

**Authors:** Ephantus J. Muturi, Ravi Kiran Donthu, Christopher J. Fields, Imelda K. Moise, Chang-Hyun Kim

**Affiliations:** 1 Crop Bioprotection Research Unit, USDA, ARS, 1815 N. University St., Peoria, IL. 61604, USA; 2 High Performance Biological Computing (HPCBio), Roy J Carver Biotechnology Center, University of Illinois at Urbana-Champaign, 1206 West Gregory Dr. Urbana, IL. 61801, USA; 3 Department of Geography and Regional Studies, University of Miami, 1300 Campo Sano Ave., Coral Gables, FL. 33146, USA; 4 Department of Public Health Sciences, Miller School of Medicine, University of Miami, USA; 5 Illinois Natural History Survey, University of Illinois at Urbana-Champaign, 1816 S. Oak St., Champaign IL 61820, USA

## Abstract

Container aquatic habitats support a specialized community of macroinvertebrates (e.g. mosquitoes) that feed on microbial communities associated with decaying organic matter. These aquatic habitats are often embedded within and around agricultural lands and are frequently exposed to pesticides. We used a microcosm approach to examine the single and combined effects of two herbicides (atrazine, glyphosate), and three insecticides (malathion, carbaryl, permethrin) on microbial communities of container aquatic habitats. MiSeq sequencing of the V4 region of both bacterial and archaeal 16S rRNA gene was used to characterize the microbial communities of indoor microcosms that were either exposed to each pesticide alone, a mix of herbicides, a mix of insecticides, or a mix of all five insecticides. Individual insecticides but not herbicides reduced the microbial diversity and richness and two insecticides, carbaryl and permethrin, also altered the microbial community structure. A mixture of herbicides had no effect on microbial diversity or structure but a mixture of insecticides or all five pesticides reduced microbial diversity and altered the community structure. These findings suggest that exposure of aquatic ecosystems to individual pesticides or their mixtures can disrupt aquatic microbial communities and there is need to decipher how these changes affect resident macroinvertebrate communities.

Container aquatic habitats, both natural (phytotelmata) and man-made (e.g. storm water catch basins, discarded tires and tins) harbor a specialized community of macroinvertebrates that is dominated by mosquitoes. These aquatic ecosystems are detritus-based and derive most of their carbon inputs from allochthonous organic matter primarily leaf litter^1^,^2^. The microbial communities associated with these aquatic ecosystems fulfil critical roles in decomposition of organic matter, nutrient cycling, and provides a critical food resource for mosquito larvae and other container-dwelling macroinvertebrate consumers^1^,^3^,^4^,^5^. Therefore, the microbial communities inhabiting these aquatic systems present a complex network of interwoven relationships between organisms of different trophic levels and their disruption can lead to many direct and indirect effects, some of which may cascade into the terrestrial environment and affect human health.

Pesticides which are comprised of insecticides, herbicides, and fungicides are a group of potentially toxic substances that are capable of disrupting the microbial structure and function in aquatic habitats. Every year, the world uses approximately 2.6 billion pounds of pesticides and the U.S. accounts for 22% of the world’s pesticide consumption^6^. Nearly 80% of these chemicals are used for agricultural purposes while a fraction of the remainder is devoted to the control of structural and public health pests^6^. Many container aquatic habitats are embedded within agricultural landscapes and may unintentionally be exposed to agricultural pesticides through spray drift, leaching and/or surface runoff. In addition, pesticides may be applied directly into these habitats to control vector mosquitoes. While most pesticides are designed to target a specific pest or particular pest group, they often extend their effects to non-target species. In fact, the frequent detection of pesticides in both surface and ground water has been associated with loss of biodiversity and detrimental effects on human and wildlife health^7^,^8^,^9^. However, despite the fundamental role of microorganisms in aquatic food webs, our understanding of how they respond to exposure to pesticides is limited.

Pesticides can cause direct toxic or beneficial effects on microbial communities similar to those reported for higher organisms. Some microbes particularly bacteria may utilize pesticides as a source of nutrients facilitating their growth and survival, while sensitive species may be impaired or decimated by pesticides^10^,^11^. These ecological alterations may trigger a cascade of indirect effects. For example, elimination or reduction of certain microbial populations by pesticides may release pesticide-tolerant microbes from competition for shared resources and thereby promote their growth and survival^5^,^12^. Similarly, some protozoan species prey on bacteria and their suppression by pesticides may facilitate survival of bacterial prey^13^. These processes may lead to dramatic shifts in microbial communities^5^,^12^,^14^ that may interfere^11^ or have little effect on microbial functions^5^,^12^,^15^.

The majority of studies that investigate the impact of pesticides on microbial communities in aquatic ecosystems have mainly focused on individual pesticides using community-level endpoints such as microbial activity, respiration, and total microbial biomass or analysis of microbial community composition using culture-dependent approaches or traditional culture-independent approaches such as quantitative real-time PCR and genetic fingerprinting^5^,^15^,^16^,^17^,^18^. These studies have provided important insights into how pesticides may affect microbial communities in aquatic systems. However, these methods are labor-intensive and may not reveal the full spectrum of microbial taxa present in a particular microbial community. In addition, aquatic ecosystems are typically exposed to a mixture of pesticides and understanding the response of microorganisms to these mixtures can improve our ability to predict the full impact of chemical disturbances on microbial processes and trophic interactions.

In this study, we used a microcosm approach to evaluate how microbial communities in container aquatic habitats respond to exposure to one of five pesticides (two herbicides and three insecticides) and their mixtures. Because testing every possible combination of the five pesticides is logistically difficult, we tested each pesticide separately and then selected a few broad combinations to determine if any of the combinations cause any detectable effects on microbial communities. This approach has proved useful in evaluating how a mixture of pollutants affect aquatic communities^19^. We tested the hypothesis that exposure to pesticides and their mixtures induces shifts in microbial diversity and community structure. The findings of this study provide an important framework for understanding how pesticide-mediated changes in microbial communities affect ecological communities in container aquatic habitats especially mosquitoes which tend to dominate in these habitats.

## Results

### Taxonomic classification of bacteria in pesticide treatments

**Figure 1:**
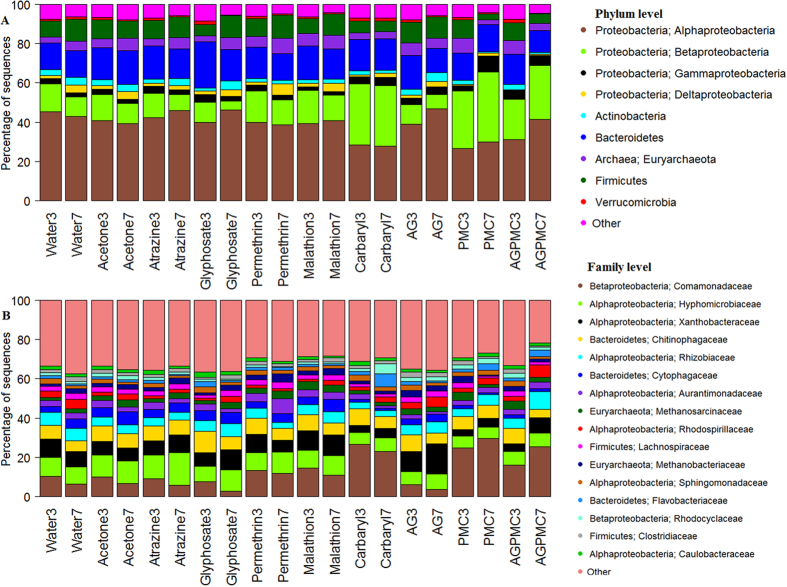
Composition of bacterial and archaeal communities at (**A**) phylum and (**B**) family level in water samples from different pesticide treatments. Taxa with abundance less than 1% and 1.6% of total sequences were pooled together as “Other” at the phylum and family level, respectively.

**Figure 2:**
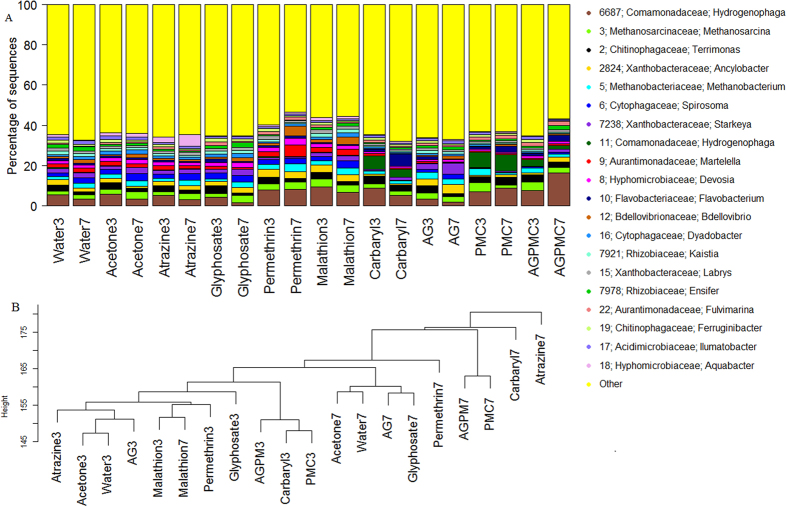
Relative abundance of top 20 OTUs (**A**) and clustering based on taxonomic composition and abundance of microbial communities (**B**) in water samples from different pesticide treatments.

Illumina sequencing of 16S rRNA amplicons yielded a total of 9.33 million sequences (Mean ± SE = 48,614 ± 1,424) across the 200 samples. After quality filtering and rarefaction at 8,702 sequences per sample, 1,653,380 sequences from 190 samples were retained and binned into 6,231 OTUs. A total of 27 phyla (2 for Archaea and 25 for bacteria) were detected across 197 families (including 6 for Archaea) but only 9 phyla had an overall abundance greater than 1% of the total sequences. The majority of sequences were from *Proteobacteria* (62.6%) including *Alphaproteobacteria* (40.0%), *Betaproteobacteria* (17.0%), *Gammaproteobacteria* (3.4%) and *Deltaproteobacteria* (2.24%) followed by *Bacteroidetes* (15.8%), and *Firmicutes* (9.07%) (Fig. 1). The relative abundance of *Alphaproteobacteria* was much lower in carbaryl, PMC, and AGPMC treatments compared to the other treatments while that of *Betaproteobacteria* was much higher in carbaryl, PMC, and AGPMC compared to the other treatments (Fig. 1). The most abundant OTUs were associated with the families *Comamonadaceae, Hyphomicrobiaceae, Xanthobacteraceae, Chitinophagaceae*, and *Rhizobiaceae* (Fig. 1). The family *Comamonadaceae* was more abundant in carbaryl, PMC, and AGPMC compared to the other treatments. Only 20 OTUs had an overall abundance greater than 1% of total sequences (Fig. 2). The five most abundant OTUs were OTU 6687-*Hydrogenophaga*, OTU 3-*Methanosarcina (Archaea: Methanobacteriaceae*), OTU 2-*Terrinonas*, OTU 2824-*Ancylobacter*, and OTU 5-*Methanobacterium (Archaea: Methanobacteriaceae*). A few OTUs were more abundant in some pesticide treatments. For example, OTU 6687 (*Hydrogenophaga*) was more abundant in treatments that contained insecticides either alone or in combination with other insecticides or herbicides while OTU 11 (*Hydrogenophaga*) was more abundant in carbaryl, PMC and AGPMC treatments (Fig. 2). There was also a tendency for microbiome of different pesticide treatments to cluster together (Fig. 2).

### Diversity of aquatic microbiota is altered by pesticide exposure

**Figure 3:**
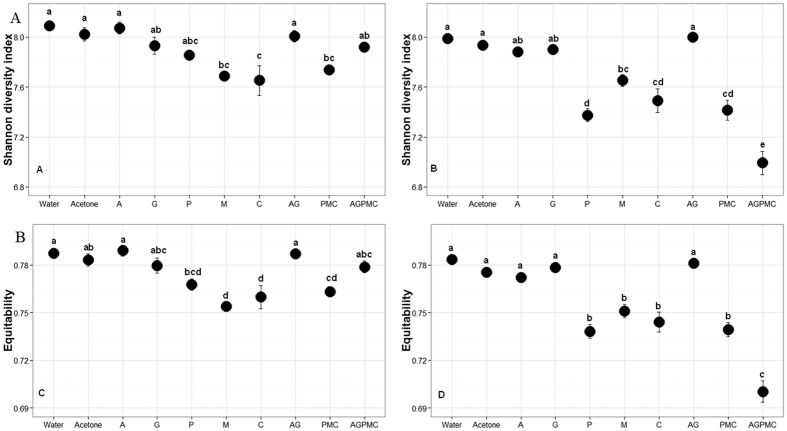
Microbial diversity and evenness in different pesticide treatments on (**A**) day 3 and (**B**) day 7. A = atrazine; G = glyphosate; P = permethrin; M = Malathion; C = carbaryl; AG = atrazine + glyphosate; PMC = permethrin + malathion + carbaryl; AGPMC = atrazine + glyphosate + permethrin + malathion + carbaryl.

Bacterial OTU diversity and evenness was significantly affected by a two-way interaction between pesticide treatment and days post treatment (Shannon index: F = 12.9, df = 9, 170, *P* < 0.0001; equitability: F = 16.9, df = 9, 170, *P* < 0.0001). Microbial diversity and evenness were consistently higher in control and herbicide treatments compared to insecticide or insecticide + herbicide combination treatments and these effects were significantly amplified on day 7 where the diversity and evenness values were highest in control and herbicide treatments, intermediate in insecticide treatments and lowest in insecticide + herbicide combination treatment (Fig. 3). For all pesticide treatments, the microbial diversity and evenness appeared to decrease over time (3 vs 7 days) with the greatest decrease occurring among AGPMC treatment (Fig. 3).

We used chao1 estimator based on OTUs abundance to determine the expected richness in each treatment (Fig. S1). On average, we detected 58.94 ± 0.24% of the expected number of OTUs for all treatments suggesting that there are undiscovered low-abundance species. The observed and predicted number of species was significantly influenced by a two-way interaction between pesticide treatments and days post exposure (observed species: F = 4.78, df = 9,170, *P* < 0.0001; chao1: F = 3.5, df = 9, 170, *P* = 0.001). At day 3 post treatment, significantly lower number of OTUs was observed in carbaryl treatment compared to controls (both water and acetone), atrazine, and permethrin treatments (Fig. S1). The number of observed OTUs was also significantly lower in PMC treatment compared to water control. On day 7, the number of observed OTUs were significantly higher in control (water and acetone), atrazine, malathion, and AG treatments compared to permethrin, PMC and AGPMC treatments.

### Microbial composition and association with environmental factors

**Figure 4:**
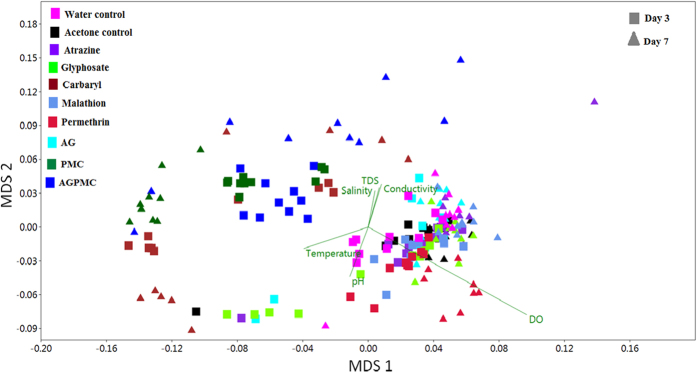
NMDS using the Bray-Curtis dissimilarity plot illustrating the microbial communities of water samples from different pesticide treatments.

**Figure 5:**
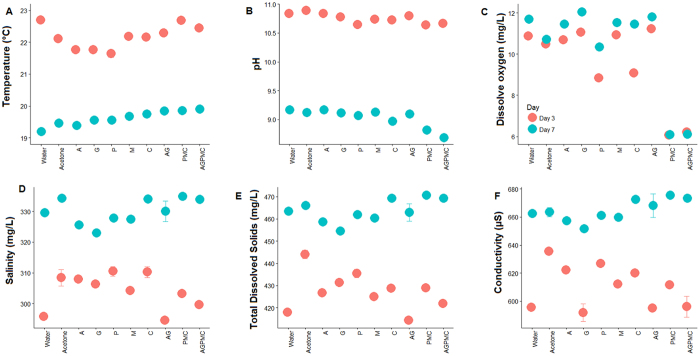
Temporal variation in the physical and chemical characteristics of the water samples (Mean ± SE) in relation to pesticide treatments. A = atrazine; G = glyphosate; P = permethrin; M = Malathion; C = carbaryl; AG = atrazine + glyphosate; PMC = permethrin + malathion + carbaryl; AGPMC = atrazine + glyphosate + permethrin + malathion + carbaryl. Error bars were too small and not visible in most data point.

The analysis of similarities (ANOSIM) indicated that the OTU composition was significantly different among pesticide treatments (R = 0.61, *P = *0.001). To visualize these differences, the taxonomic abundance profiles were used to compute a Bray-Curtis similarity matrix coordinated into two dimensions by using NMDS (Fig. 4). This analysis revealed clear separation of microbial communities of carbaryl, PMC, and AGPM and other treatments (Fig. 4). Carbaryl, PMC, and AGPM samples were also more heterogeneous compared to other treatments and dissolved oxygen contributed most to the separation. Carbaryl, PMC, and AGPM treatments were associated with low dissolved oxygen compared to other treatment. Although the other environmental variables did not contribute much to the observed structuring of microbial communities, they varied significantly between pesticide treatments, day of sample collection, and their interaction, with pH contributing most to the observed variation (Table 2; Fig. 5).

SIMPER analysis highlighted 254 OTUs that were primarily responsible for observed differences between pesticide treatments. Of these OTUs, there were 6 OTUs that contributed an average dissimilarity equal to or greater than 1% between pesticide treatments (Table S1). Together, OTU 11 (*Hydrogenophaga*) and OTU 2 (*Terrimonas*) contributed 8.2% of observed differences between treatments and were more abundant in carbaryl, PMC and AGPMC treatments. OTU 3 (*Methanosarcina*) accounted for 3.2% of observed variation and was more abundant in permethrin, malathion, PMC, and AGPMC treatments. OTU 6 (*Spirosoma*) was less abundant in carbaryl, PMC, AGPMC treatments while OTU 9 (*Alphaproeteobacteria*) was less abundant in carbaryl, glyphosate, AG, PMC and AGPMC. OTU 5 was more abundant in permethrin, malathion, and AG treatments.

## Discussion

In this study, we examined how a suite of commonly used pesticides both separately and as mixtures affect the microbial communities of container aquatic habitats. Our results show that low concentrations of separate and combined pesticides can reduce microbial diversity and induce shifts in microbial community structure in these aquatic systems. These effects were dependent both on pesticide identity and day of sampling. Individual insecticides reduced the microbial diversity, and two of the three insecticides (carbaryl and permethrin) induced shifts in microbial community structure. In contrast, individual herbicides or their combinations had no effect on either microbial diversity or community structure. The mixtures of the three insecticides or all five pesticides reduced the microbial diversity and caused major shifts in microbial community structure. In addition, the effect of pesticides on microbial diversity particularly the effects of insecticide mixtures or mixtures of all five pesticides were more pronounced on day 7 compared to day 3. However, due to the nature of our experimental design, we could not determine whether the effect of pesticide mixtures were either due to synergistic effects among pesticides or due to greater overall concentration of pesticides in the microcosms.

Each of the five pesticides examined in this study has been shown to affect microbial communities in aquatic habitats^17^,^18^,^20^,^21^,^22^,^23^. However, this study is the first to provide a comprehensive analysis of individual and combined effects of pesticides on microbial composition and structure in aquatic habitats. Changes in the diversity and structure of microbial communities following exposure to pesticides may result from toxic effects of pesticides on some microbial groups and proliferation of tolerant species due to reduced competition for space and available nutrients^12^. Some microbes are also capable of utilizing pesticides as an energy source and may benefit from pesticide exposure^10^. Pesticides also may affect microbial communities indirectly by altering the physical and chemical characteristics of the aquatic habitats which may suppress some microbial species while stimulating growth and survival of others^24^. Our study was not designed to determine which of these mechanisms were responsible for observed changes in microbial diversity and composition following exposure to pesticides. However, some pesticide treatments including carbaryl, a mixture of the three insecticides, and a mixture of all five pesticides were associated with low dissolved oxygen concentrations that may have affected microbial communities. In fact, the three bacterial taxa that were strongly associated with carbaryl, PMC and AGPMC treatments including OTU 2 (*Terrimonas*), OTU 3 (*Methanosarcina*), and OTU 11 (*Hydrogenophaga*) are either known to thrive at low oxygen concentrations or to degrade pesticides and may have benefited from pesticide exposure^25^,^26^,^27^.

The lack of significant effects of atrazine and glyphosate on microbial communities was not expected since our previous study using a similar experimental design had revealed that both herbicides can induce shifts in microbial community structure^23^. However, this earlier study used much lower herbicide concentrations (1 mg/L) compared to the current study (20 mg/L) and focused on the abundance of five bacterial phyla as opposed to overall bacterial composition at lowest possible taxonomic unit in the current study. Water samples for this previous study were also collected 2 days after adding the pesticides compared to 3 and 7 days after pesticide additions in the current study. These findings suggest that analysis of a few bacterial taxa at higher taxonomic levels may not provide an accurate measure of how pesticides may affect microbial communities in aquatic systems. It is also possible that microbial communities responded differently to varying concentrations of each of the two herbicides as reported previously^20^,^22^.

Interestingly, we found that the effects of all five pesticides on microbial diversity were not clearly evident on day 3 as they were on day 7. These findings suggest that atrazine and glyphosates may delay the onset of negative effects of insecticides on microbial communities. We did not examine the mechanism underlying these findings but generally interactions between pesticides can affect six processes that may influence their toxicity including bioavailability, uptake, internal transportation, metabolism, binding at the target site, and excretion^28^. Future studies should examine which of these processes are responsible for our findings. In addition, we neither quantified the rate of pesticide breakdown in our microcosms nor how long the effects of pesticides on microbial communities persisted. We also focused on bacterial and archaeal communities yet other taxa including fungi, protozoa and algae are a major component of aquatic microbial communities. Moreover, our experiment was conducted in simple indoor microcosms using NRO infusion yet in nature, container aquatic habitats are exposed to pesticides in the presence of different types of leaf mixtures and other biotic and abiotic factors that were not included in this study. Future studies addressing these knowledge gaps will reveal further insights into the impact of pesticides on microbial communities.

Our findings suggest that short-term exposure to low concentrations of pesticides may have profound effects on key ecological processes in container aquatic ecosystems. Microbial communities are key mediators of critical ecosystem processes such as decomposition of organic matter, nutrient cycling, and detoxification of organic pollutants^1^,^3^,^4^,^5^. Pesticide-mediated changes in microbial diversity and community structure can disrupt these ecosystem functions if the pesticide-tolerant microorganisms are unable to compensate for loss of function associated with pesticide-sensitive microbes^12^,^24^,^29^. In addition, microbial communities form the base of aquatic food webs, and their disruption may alter the quality and quantity of food resource available for container-dwelling macroinvertebrates. These changes may affect individual growth, survival, and reproduction and may then be manifested both at the population (e.g. population growth) and community level (e.g. food web structure, species richness). Mosquito immatures tend to be the dominant macroinvertebrates in container aquatic habitats and utilize aquatic microbes as a direct food resource^1^,^30^. These microbes also produce chemical compounds that aid gravid female mosquitoes to select high quality aquatic habitats for oviposition, and stimulate egg hatch^31^,^32^. Thus pesticides may have important implications on ecology and behavior of mosquitoes if they promote or disrupt the specific microbial taxa that mediate these processes.

In summary, we observed that the bacterial and archaeal communities of container aquatic habitats shifted in response to disturbance by pesticides or their mixtures. These changes seemed to be mainly driven by insecticides but not herbicides. These findings provide the basis for understanding how pesticide-mediated changes in aquatic microbial communities may affect aquatic communities as well as key ecological processes such as decomposition, nutrient cycling, and bioremediation. The findings also are important for understanding how anthropogenic chemical contaminants affect human health because container aquatic habitats are dominated by mosquitoes which transmit a wide variety of devastating and life threatening human pathogens.

## Materials and Methods

### Experimental design

The experiment was conducted using microcosms established with Northern Red Oak (NRO) infusion. The infusion was prepared by mixing 500 g of senescent NRO leaves with 122 L of tap water and fermenting for 11 days at room temperature. Experimental microcosms consisted of 400 mL tri-pour beakers filled with 200 mL NRO infusion. The treatments were composed of a negative control (water), a vehicle control (acetone), one of two herbicides applied separately (atrazine (A), and glyphosate (G)), one of three insecticides applied separately (permethrin (P), malathion (M), and carbaryl (C)), a mix of the two herbicides (AG), a mix of the three insecticides (PMC), and a mix of all five pesticides (AGPMC, Table 1). These pesticides were chosen because they are among the most widely used and are common contaminants of aquatic ecosystems^33^,^34^. Atrazine controls broadleaf weeds by inhibiting photosystem II while glyphosate acts by inhibiting amino acid synthesis. Permethrin is a pyrethroid insecticide that kills insects by prolonging sodium channel activation. Malathion (organophosphate) and carbaryl (carbamate) insecticides act by inhibiting acetylcholine esterase. Each treatment was replicated 10 times for a total of 100 containers.

On day 0, the microcosms were treated with either 0.4 mL of water control or an equal volume of 10,000 mg/L of each of the target pesticides for a nominal concentration of 20 mg/L for each pesticide. Thus the concentrations of pesticide mixtures were either 2 (for herbicides), 3 (for insecticides), or 5 (for all pesticides combined) times higher than those of individual pesticides. A vehicle control (acetone control) was included in which 2 mL of acetone was added. This amount is the same as the amount of acetone that was added to treatments receiving all five pesticides. Because the goal of this study was to examine the separate and combined effects of different pesticides on microbial communities, the choice of nominal concentrations of 20 mg/L was arbitrary. To maintain equal volume of final solution for all containers, a desired volume of NRO infusion was removed from each container before adding the target pesticide treatment(s). For example, 0.4 mL of NRO infusion was removed from individual pesticide treatment containers prior to adding 0.4 mL of target pesticide. Similarly, 0.8 mL, 1.2 mL, and 2 mL of NRO infusion were removed from treatments with two, three, and five pesticide mixtures, respectively before adding 0.4 mL of each of the target pesticides. Finally, 2 mL of NRO infusion was removed in acetone control before adding 2 mL of acetone. The microcosms were held at room temperature throughout the study period. Although container aquatic habitats are primarily dominated by mosquitoes, we did not quantify the response of microbial communities to pesticides in the presence of mosquito larvae. The pesticide doses used in this study are lethal to mosquitoes and reflect the typical doses that would be associated with vector control activities, spray drifts, or runoffs from nearby agricultural farm that may occur immediately after pesticide application.

On day 3 and day 7 after adding the pesticides, 15 mL aliquots of water samples were taken from each container and stored at −80 °C until further processing. Immediately after collection of the water samples, the physical and chemical characteristics of each microcosm including temperature, pH, salinity, dissolved oxygen, total dissolved solids, and conductivity were measured using ExStik II^®^ probes (Extech instruments, Nashua, NH).

### DNA extraction and 16S rRNA library preparation

The water samples were retrieved from the freezer, thawed, and centrifuged at 5000 × g for 20 minutes. The resulting pellet was resuspended in Bead Solution of Ultraclean^®^ Soil DNA isolation kit (MoBio, Carlsbad, CA) and total DNA was extracted using Ultraclean^®^ Soil DNA isolation kit (MoBio). DNA was quantified using NanoDrop 1000 (Thermo Scientific, Pittsburg, PA) and its quality assessed using Agilent 2100 Bioanalyzer (Agilent Technologies, Santa Clara, CA). The V4 region of both bacterial and archaeal 16S rRNA gene was amplified using the primer set forward, 5′–GTGCCAGCMGCCGCGGTAA–3′ and reverse, 5′–GGACTACHVGGGTWTCTAAT–3′^35^ and sequenced using Illumina MiSeq Bulk v3 platform at the W. M. Keck Center for Comparative and Functional Genomics at the University of Illinois at Urbana-Champaign as previously described^36^.

In brief, all DNA samples were measured on a Qubit (Life Technologies) using High Sensitivity DNA Kit and diluted to 2 ng/μl. A master mix containing 0.5 μl -10X FastStart Reaction Buffer without MgCl_2_, 0.9 μl -25 mM MgCl_2_, 0.25 μl -DMSO, 0.1 μl -10 mM PCR grade Nucleotide Mix, 0.05 μl -5 U/μl FastStart High Fidelity Enzyme Blend, 0.25 μl -20X Access Array Loading Reagent, and 0.95 μl -water was prepared using the Roche High Fidelity Fast Start Kit and 20X Access Array loading reagent and aliquoted into 48 well PCR plates along with 1 μl DNA sample and 1 μl Fluidigm Illumina linkers (V3-V5-F357: ACACTGACGACATGGTTCTACA and V3-V5-R926:TACGGTAGCAGAGACTTGGTCT) with unique barcode. In a separate plate, primer pairs were prepared and aliquoted. 20X primer solutions were prepared by adding 2 μl of each forward and reverse primer, 5 μl of 20X Access Array Loading Reagent and water to a final volume of 100 μl.

Four μl of sample was loaded in the sample inlets and 4 μl of primer loaded in primer inlets of a previously primed Fluidigm 48.48 Access Array IFC. The IFC was placed in an AX controller (Fluidigm Corp.) for microfluidic loading of all primer/sample combinations. Following the loading stage, the IFC plate was loaded on the Fluidigm Biomark HD PCR machine and samples were amplified using the following Access Array cycling program without imaging: 50 °C for 2 minutes (1 cycle), 70 °C for 20 minutes (1 cycle), 95 °C for 10 minutes (1 cycle), followed by 10 cycles at 95 °C for 15 seconds, 60 °C for 30 seconds, and 72 °C for 1 minute, 2 cycles at 95 °C for 15 seconds, 80 °C for 30 seconds, 60 °C for 30 seconds, and 72 °C for 1 minute, 8 cycles at 95 °C for 15 seconds, 60 °C for 30 seconds, and 72° for 1 minute, 2 cycles at 95 °C for 15 seconds, 80 °C for 30 seconds, 60 °C for 30 seconds, and 72 °C for 1 minute, 8 cycles at 95 °C for 15 seconds, 60 °C for 30 seconds, and 72 °C for 1 minute, and 5 cycles at 95 °C for 15 seconds, 80 °C for 30 seconds, 60 °C for 30 seconds, and 72 °C for 1 minute. The PCR product was transferred to a new 96 well plate, quantified on a Qubit fluorimeter (Thermo-Fisher) and stored at −20 °C. All samples were run on a Fragment Analyzer (Advanced Analytics, Ames, IA) and amplicon regions and expected sizes confirmed. Samples were then pooled in equal amounts according to product concentration. The pooled products were size selected on a 2% agarose E-gel (Life Technologies) and extracted from the isolated gel slice with QIAquick gel extraction kit (QIAGEN). Cleaned size selected products were run on an Agilent Bioanalyzer to confirm appropriate profile and determination of average size. The final library pool was spiked with 10% non-indexed PhiX control library (Illumina^®^) and sequenced using Illumina^®^ MiSeq^®^ V3 Bulk system. The libraries were sequenced from both ends of the molecules to a total read length of 300 nt from each end. Cluster density was 964 k/mm2 with 85.9% of clusters passing filter.

### OTU picking and taxonomy assignment

De-multiplexed forward (read 1) and reverse reads (read 2) obtained from the sequencing facility were processed using the IM-TORNADO v2.0.3.2 pipeline^37^. Trimmomatic program was used to trim the forward and the reverse primers from the 5′ end of read 1 and read 2 respectively, and the adaptor sequences from the 3′ end of the reads^38^; http://www.usadellab.org/cms/? page=trimmomatic). Since paired reads from the V4 region overlap, PEAR 0.9.2 was used to merge the trimmed reads using default parameters^39^; http://sco.h-its.org/exelixis/web/software/pear/index.html). Merged reads were processed using IM-TORNADO pipeline^37^ to trim low quality bases, remove reads with ambiguous bases, and perform *de novo* OTU picking with sequences containing 150 or more bases. Because IM-TORNADO requires both R1 reads and R2 reads as input, stitched reads obtained from PEAR were considered as R1 reads and the reverse complement of R1 reads were treated as R2 reads for each sample. Reads were de-replicated building clusters of reads with 100% similarity and annotated with cluster size. To ensure the use of high quality reads when assigning OTU representation, singletons and reads shorter than the cutoff length were discarded. Reads were sorted by cluster size and processed in USEARCH using the UPARSE algorithm to find the OTU representatives using *de novo* OTU picking strategy. During this step, chimeric reads are also removed resulting in a set of OTU representatives of very high sequence quality^40^. The operational taxonomic units (OTUs) were picked and identified at 97% similarity using the Ribosomal Database Project (RDP) version 10 as the reference database^41^. IM-TORNADO performs taxonomy assignment of the OTUs by running classify. seqs function of mothur^42^. We included only annotations with a bootstrap confidence greater than 50%. In a single run, IM-TORNADO generates outputs for R1 data only, R2 data only and paired end data. Output files related to R1 data were used for downstream analysis.

### Sequencing data submission

All sequencing raw datasets have been deposited in the National Center for Biotechnology Information (NCBI) Sequence Read Archive (SRA) database (https://www.ncbi.nlm.nih.gov/sra/) with the BioProject accession number PRJNA374734.

### Statistical analysis

Prior to statistical analysis, OTUs with less than 6 sequences were discarded because rare sequences likely represents random sequencing errors and may overestimate the overall diversity^43^. The number of sequences varied markedly among individual microcosms (range 4-147,197) and thus read depth was rarefied to 8,702 reads per microcosm to standardize the sampling effort. This process led to loss of 1 or 2 replicates in some treatments (Table 1). Alpha diversity metrics including Shannon diversity index, observed species, chao1, and evenness were generated in QIIME and analysis of variance (ANOVA) with Tukey adjustments was used to test the effect of pesticides treatments on these indices using R version 3.2.3 statistical package (https://cran.r-project.org/bin/windows/base/old/3.2.3/). Analysis of similarities (ANOSIM) with 999 permutations was used to test whether microbial communities from the same pesticide treatments were more similar than microbial communities from different pesticide treatments. This was accomplished using vegan package in R^44^. Taxonomic abundance profiles were used to compute a Bray-Curtis similarity matrix coordinated into two dimensions using non-metric multidimensional scaling (NMDS) considering OTU distribution and water chemistry variables. To identify OTUs that were primarily responsible for observed differences between pesticide treatments, similarity percentage (SIMPER) analysis was used. Both NMDS and SIMPER analysis were conducted using the software PAST (http://folk.uio.no/ohammer/past/)^45^.

## Additional Information

**How to cite this article**: Muturi, E. J. *et al*. Effect of pesticides on microbial communities in container aquatic habitats. *Sci. Rep.* **7**, 44565; doi: 10.1038/srep44565 (2017).

**Publisher's note:** Springer Nature remains neutral with regard to jurisdictional claims in published maps and institutional affiliations.

## Supplementary Material

Supplementary Information

## Figures and Tables

**Table 1:** Pesticide treatments used in the current study.

Pesticide treatment	Labels	Number of Replicates		
		Initial	Day 3 final	Day 7 final
Water control	Water	10	10	9
Acetone	Acetone	10	10	10
Atrazine (A)	A	10	10	9
Glyphosate (G)	G	10	10	9
Permethrin (P)	P	10	10	8
Malathion (M)	M	10	10	9
Carbaryl (C)	C	10	9	9
A + G	AG	10	10	9
P + M + C	PMC	10	10	9
A + G + P + M + C	AGPMC	10	10	10

**Table 2:** Multivariate ANOVA for the effect of pesticides and day of sample collection on the physical and chemical characteristics of the water samples.

				Standardized canonical coefficients					
Variable	Pillai s Trace	df	P	Temperature	pH	Salinity	TDS	Conductivity	Dissolved oxygen
Pesticide (P)	3.19	54, 1080	<0.0001	−1.00	15.28	−0.19	−0.66	−0.58	5.36
Day (D)	1.00	6, 175	<0.0001	3.18	36.67	−0.26	−1.88	0.37	−1.83
P x D	2.92	54, 1080	<0.0001	2.99	33.96	−0.82	−2.12	0.25	0.11

## References

[b1] Walker E. D., Lawson D. L., Merritt R. W., Morgan W. T. & Klug M. J. Nutrient dynamics, bacterial populations, and mosquito productivity in tree hole ecosystems and microcosms. Ecology 72, 1529–1546 (1991).

[b2] Kitching R. L. Food webs and container habitats: the natural history and ecology of phytotelmata. Cambridge, UK. Cambridge University Press (2000).

[b3] Walker E. D. & Merritt R. W. The significance of leaf detritus to mosquito (Diptera, Culicidae) productivity from treeholes. Environ.Entomol. 17, 199–206 (1988).

[b4] Mathuriau C. & Chauvet E. Breakdown of leaf litter in a neotropical stream. J. N. Am. Benthol. Soc. 21, 384–396 (2002).

[b5] Widenfalk A., Bertilsson S., Sundh I. & Goedkoop W. Effects of pesticides on community composition and activity of sediment microbes - responses at various levels of microbial community organization. Environ. Pollut. 152, 576–584 (2008). 17822816 10.1016/j.envpol.2007.07.003

[b6] Grube A., Donaldson D., Kiely T. & Wu L. Pesticide industry sales and usage: 2006 and 2007 market estimates. Biological and Economic Analysis Division, U.S. Environmental Protection Agency (2011).

[b7] Horrigan L., Lawrence R. S. & Walker P. How sustainable agriculture can address the environmental and human health harms of industrial agriculture. Environ. Health Perspect. 110, 445–456 (2002). 12003747 10.1289/ehp.02110445 PMC1240832

[b8] Bridges C. M. Long-term effects of pesticide exposure at various life stages of the southern leopard frog (Rana sphenocephala). Arch. Environ. Contam. Toxicol. 39, 91–96 (2000). 10790507 10.1007/s002440010084

[b9] Davidson C., Shafer H. B. & Jennings M. R. Declines of of California red-llegged frog: climate, UV-B, habitat, and pesticide hypotheses. Ecol. App. 11, 464–479 (2001).

[b10] Russell R. J. . The evolution of new enzyme function: lessons from xenobiotic metabolizing bacteria versus insecticide-resistant insects. Evol. Appl. 4, 225–248 (2011). 25567970 10.1111/j.1752-4571.2010.00175.x PMC3352558

[b11] DeLorenzo M. E., Scott G. I. & Ross P. E. Toxicity of pesticides to aquatic microorganisms: A review. Environ. Toxicol. Chem. 20, 84–98 (2001). 11351418 10.1897/1551-5028(2001)020<0084:toptam>2.0.co;2

[b12] Johnsen K., Jacobsen C. S., Torsvik V. & Sorensen J. Pesticide effects on bacterial diversity in agricultural soils - a review. Biol. Fert. Soils 33, 443–453 (2001).

[b13] Staley Z. R., Harwood V. J. & Rohr J. R. A synthesis of the effects of pesticides on microbial persistence in aquatic ecosystems. Crit. Rev. Toxicol. 45, 813–836 (2015). 26565685 10.3109/10408444.2015.1065471 PMC4750050

[b14] Lupwayi N. Z. . Changes in functional structure of soil bacterial communities due to fungicide and insecticide applications in canola. Agric. Ecosyst. Environ. 130, 109–114 (2009).

[b15] Widenfalk A., Svensson J. M. & Goedkoop W. Effects of the pesticides captan, deltamethrin, isoproturon, and pirimicarb on the microbial community of a freshwater sediment. Environ. Toxicol. Chem. 23, 1920–1927, doi: 10.1897/03-345 (2004). 15352481

[b16] Widenfalk A., Lundqvist A. & Goedkoop W. Sediment microbes and biofilms increase the bioavailability of chlorpyrifos in Chironomus riparius (Chironomidae, Diptera). Ecotoxicol. Environ. Safety 71, 490–497 (2008). 18093655 10.1016/j.ecoenv.2007.10.028

[b17] Downing A. L., DeVanna K. M., Rubeck-Schurtz C. N., Tuhela L. & Grunkemeyer H. Community and ecosystem responses to a pulsed pesticide disturbance in freshwater ecosystems. Ecotoxicol. 17, 539–548 (2008). 10.1007/s10646-008-0211-3 18437563

[b18] Downing H. F. . Effects of the agricultural pesticides atrazine, chlorothalonil, and endosulfan on South Florida microbial assemblages. Ecotoxicol. 13, 245–260 (2004). 10.1023/b:ectx.0000023569.46544.9f 15217248

[b19] Relyea R. A. A cocktail of contaminants: how mixtures of pesticides at low concentrations affect aquatic communities. Oecologia 159, 363–376 (2009). 19002502 10.1007/s00442-008-1213-9

[b20] DeLorenzo M. E., Lauth J., Pennington P. L., Scott G. I. & Ross P. E. Atrazine effects on the microbial food web in tidal creek mesocosms. Aquatic Toxicol. 46, 241–251 (1999).

[b21] Weber F. H. & Rosenberg F. A. Interactions of carbaryl with estuarine bacterial communities. Microb. Ecol. 10, 257–269 (1984). 24221147 10.1007/BF02010939

[b22] Stachowski-Haberkorn S. . Impact of Roundup on the marine microbial community, as shown by an *in situ* microcosm experiment. Aquatic Toxicol. 89, 232–241 (2008). 10.1016/j.aquatox.2008.07.004 18760491

[b23] Muturi E. J., Orindi B. O. & Kim C. H. Effect of leaf type and pesticide exposure on abundance of bacterial taxa in mosquito larval habitats. PLoS ONE 8, e71812, (2013). 23940789 10.1371/journal.pone.0071812 PMC3733839

[b24] Schafer R. B. . Effects of pesticide toxicity, salinity and other environmental variables on selected ecosystem functions in streams and the relevance for ecosystem services. Sci. Total Environ. 415, 69–78 (2012). 21802709 10.1016/j.scitotenv.2011.05.063

[b25] Aislabie J. & Lloyd-Jones G. A review of bacterial degradation of pesticides. Aust. J. Soil Res. 33, 925–942 (1995).

[b26] Singh B. K. & Walker A. Microbial degradation of organophosphorus compounds. FEMS Microbiol. Rev. 30, 428–471 (2006). 16594965 10.1111/j.1574-6976.2006.00018.x

[b27] Lovecka P., Janu P., Uhlik O., Mackova M. & Demnerova K. Environmental impact of organochlorinated pesticides. Environ. Eng. Manag. J. 11, S103 (2012).

[b28] Cedergreen N. Quantifying synergy: a systematic review of mixture toxicity studies within environmental toxicology. PLoS One 9, e96580 (2014). 24794244 10.1371/journal.pone.0096580 PMC4008607

[b29] Schafer R. B. . Effects of pesticides on community structure and ecosystem functions in agricultural streams of three biogeographical regions in Europe. Sci. Total Environ. 382, 272–285 (2007). 17555800 10.1016/j.scitotenv.2007.04.040

[b30] Merritt R. W., Dadd R. H. & Walker E. D. Feeding behavior, natural food, and nutritional relationships of larval mosquitoes. Annu. Rev. Entomol. 37, 349–376 (1992). 1347208 10.1146/annurev.en.37.010192.002025

[b31] Ponnusamy L., Boroczky K., Wesson D. M., Schal C. & Apperson C. S. Bacteria stimulate hatching of yellow fever mosquito eggs. PLoS ONE 6, e24409 (2011). 21915323 10.1371/journal.pone.0024409 PMC3167859

[b32] Ponnusamy L. . Identification of bacteria and bacteria-associated chemical cues that mediate oviposition site preferences by *Aedes aegypti*. Proc. Natl. Acad. Sci. USA 105, 9262–9267 (2008). 18607006 10.1073/pnas.0802505105 PMC2443818

[b33] Gilliom R. J. Pesticides in U.S. streams and groundwater. US Geological Survey circular 3409 (2007). 10.1021/es072531u 17547156

[b34] Battaglin W. A., Meyer M. T. & Dietze J. E. Widespread occurrence of Glyphosate and its degradation product (AMPA) in U.S. soils, surface water, groundwater, and precipitation, 2001–2009. Amer. Geophys Union, abstract #H44A-08 (2011).

[b35] Wu L. Y. . Phasing amplicon sequencing on Illumina Miseq for robust environmental microbial community analysis. BMC Microbiol. 15, 125 (2015). 26084274 10.1186/s12866-015-0450-4 PMC4472414

[b36] Muturi E. J., Kim C. H., Bara J., Bach E. M. & Siddappaji M. H. *Culex pipiens* and *Culex restuans* mosquitoes harbor distinct microbiota dominated by few bacterial taxa. Parasit. Vectors 9, 18 (2016). 26762514 10.1186/s13071-016-1299-6 PMC4712599

[b37] Jeraldo P. . IM-TORNADO: a tool for comparison of 16S reads from paired-end libraries. PLoS One 9, e114804 (2014). 25506826 10.1371/journal.pone.0114804 PMC4266640

[b38] Lohse M. . RobiNA: a user-friendly, integrated software solution for RNA-Seq-based transcriptomics. Nucleic Acids Res. 40, W622–627 (2012). 22684630 10.1093/nar/gks540 PMC3394330

[b39] Zhang J., Kobert K., Flouri T. & Stamatakis A. PEAR: a fast and accurate Illumina Paired-End reAd mergeR. Bioinformatics 30, 614–620 (2014). 24142950 10.1093/bioinformatics/btt593 PMC3933873

[b40] Edgar R. C. UPARSE: highly accurate OTU sequences from microbial amplicon reads. Nature Meth. 10, 996–998 (2013). 10.1038/nmeth.2604 23955772

[b41] Cole J. R. . Ribosomal Database Project: data and tools for high throughput rRNA analysis. Nucleic Acids Res 42, D633–642 (2014). 24288368 10.1093/nar/gkt1244 PMC3965039

[b42] Schloss P. D. . Introducing mothur: open-source, platform-independent, community-supported software for describing and comparing microbial communities. Appl. Environ. Microbiol. 75, 7537–7541 (2009). 19801464 10.1128/AEM.01541-09 PMC2786419

[b43] Boissiere A. . Midgut microbiota of the malaria mosquito vector Anopheles gambiae and interactions with *Plasmodium falciparum* infection. PLoS Pathog. 8, e1002742, (2012). 22693451 10.1371/journal.ppat.1002742 PMC3364955

[b44] Oksanen J. . vegan: Community ecology package. https://CRAN.R-project.org/package=vegan (2013).

[b45] Hammer O., Harper D. A. T. & Ryan P. D. PAST: Paleontological statistics software package for education and data analysis. Paleontologia Electronica 4, 4–9 (2001).

