# Supplementary material for: Effect of pesticides on microbial communities in container aquatic habitats
Source: Sci Rep. 2017 Mar 16;7:44565. doi: 10.1038/srep44565 (PMC5353589; doi:10.1038/srep44565)

**Effect of pesticides on microbial communities in mosquito aquatic habitats**

Ephantus J. Muturi^1*^, Ravi Kiran Donthu^2^, Christopher J. Fields^2^, Imelda K. Moise^3,4^, Chang-Hyun Kim^5^

^1^Crop Bioprotection Research Unit, USDA, ARS, 1815 N. University St., Peoria, IL. 61604.

^2^High Performance Biological Computing (HPCBio), Roy J Carver Biotechnology Center, University of Illinois at Urbana-Champaign, 1206 West Gregory Dr. Urbana, IL 61801.

^3^Department of Geography and Regional Studies, University of Miami, 1300 Campo Sano Ave., Coral Gables, FL. 33146.

^4^Department of Public Health Sciences, Miller School of Medicine, University of Miami.

^5^Illinois Natural History Survey, University of Illinois at Urbana-Champaign, 1816 S. Oak St., Champaign IL 61820.

*Corresponding Author

Email: [Ephantus.Muturi@ars.usda.gov](mailto:Ephantus.Muturi@ars.usda.gov).

Phone: 309-681-6194

**Table S1: SIMPER analysis results comparing water samples from different pesticide treatments.** The Bray-Curtis average dissimilarity between pesticide treatments was greater than 1% for the six OTUs. A = atrazine; G = glyphosate; P = permethrin; M = malathion; C = carbaryl; AG = a mixture of atrazine and glyphosate; PMC = a mixture of permethrin, glyphosate and carbaryl; AGPMC = a mixture of all pesticides.

| OTU | Taxon | Average  dissimilarity | Percentage  contribution | Cumulative percentage | Water | Acetone | A | G | P | M | C | AG | PMC | AGPMC |
| --- | --- | --- | --- | --- | --- | --- | --- | --- | --- | --- | --- | --- | --- | --- |
| 11 | *Hydrogenophaga* | 2.10 | 5.45 | 5.45 | 0.48 | 0.34 | 0.39 | 0.27 | 0.87 | 0.52 | 8.31 | 0.29 | 12.40 | 4.31 |
| 3 | *Methanosarcina* | 1.23 | 3.18 | 8.63 | 3.15 | 4.73 | 3.71 | 3.81 | 5.29 | 6.66 | 3.09 | 4.61 | 5.03 | 5.85 |
| 6 | *Spirosoma* | 1.20 | 3.12 | 11.76 | 3.70 | 4.95 | 4.14 | 4.81 | 4.25 | 5.00 | 0.67 | 3.38 | 0.73 | 1.28 |
| 5 | *Methanobacterium* | 1.06 | 2.75 | 14.51 | 3.04 | 3.49 | 3.35 | 2.94 | 4.89 | 4.52 | 2.44 | 4.90 | 3.28 | 3.33 |
| 2 | *Terrimonas* | 1.04 | 2.70 | 17.20 | 3.87 | 4.06 | 3.67 | 4.44 | 4.41 | 4.46 | 5.78 | 4.42 | 5.49 | 5.41 |
| 9 | *Alphaprotobacteria* | 1.04 | 2.68 | 19.89 | 3.34 | 3.36 | 3.07 | 1.84 | 6.16 | 4.59 | 1.32 | 1.05 | 1.21 | 1.40 |

**Figure S1: Observed and predicted number of OTUs in different pesticide treatments on A) day 3 and B) day 7.** A = atrazine; G = glyphosate; P = permethrin; M = Malathion; C = carbaryl; AG = atrazine + glyphosate; PMC = permethrin + malathion + carbaryl; AGPMC = atrazine + glyphosate + permethrin + malathion + carbaryl.


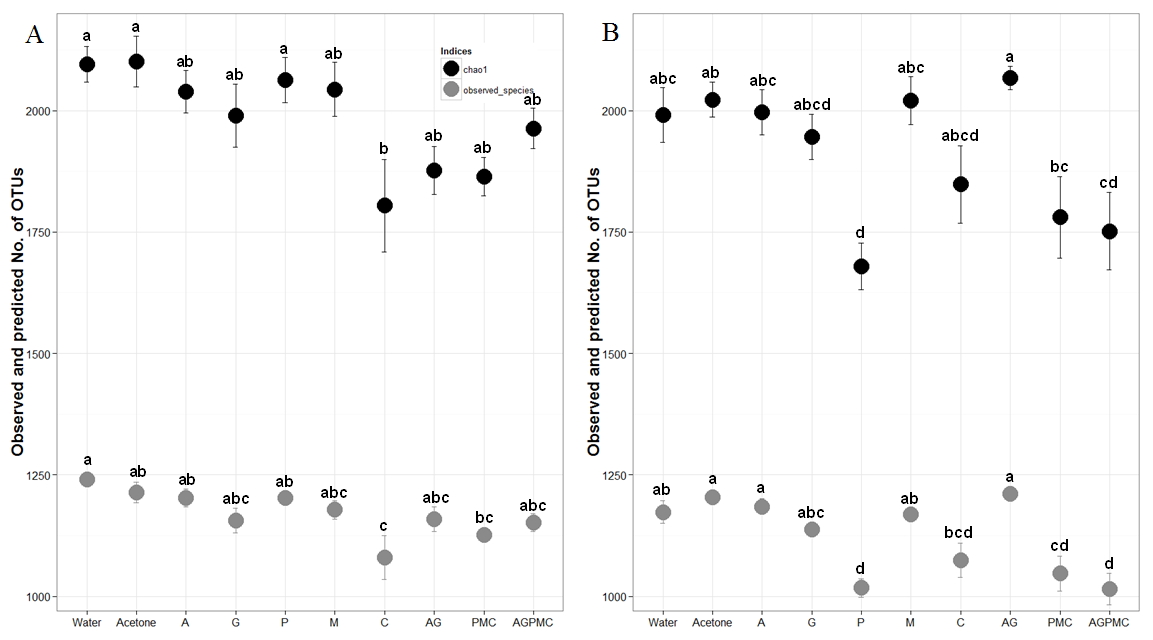

Supplement: Supplementary Information [file srep44565-s1.docx]
